# Effects of Tarragon Hydrodistillate and Essential Oil on Aquatic Ecosystems

**DOI:** 10.3390/toxics13080668

**Published:** 2025-08-08

**Authors:** Andrijana Pujicic, Bianca-Vanesa Agachi, Constantina-Bianca Vulpe, Adriana Isvoran

**Affiliations:** 1 Department of Biology, West University of Timisoara, Blvd. V Parvan 4, 300223 Timisoara, Romania; andrijana.pujicic@e-uvt.ro; 2 Department of Biology, Advanced Environmental Research Institute, Oituz, 4, 300086 Timisoara, Romania; constantina.vulpe@e-uvt.ro

**Keywords:** *Artemisia dracunculus*, ecotoxicity, growth inhibition test, biochemical assay, aquatic organism risk assessment

## Abstract

Tarragon extracts, especially from *Artemisia dracunculus*, have shown their potential as natural pesticides and can harm aquatic ecosystems. In addition, waste from tarragon essential oil production can also contribute to aquatic pollution if not properly managed. In this study, a hydrodistillate and a commercial tarragon essential oil were considered to evaluate their effects on aquatic ecosystems. A growth inhibition test was performed using *Lemna minor* to evaluate the potential ecotoxicity of tarragon extracts, and a biochemical test was performed to investigate the potential effects of the lowest volume of oil, which did not cause any visible impact on this organism. The results showed that the hydrodistillate did not show toxic effects on *L. minor*, but the essential oil demonstrated potential ecotoxicity, with volumes of 0.5 µL and above leading to percentage reductions in frond numbers of 50% and higher. The biochemical assay revealed no significant differences between the negative control and the lowest volume of oil tested, suggesting the absence of biochemical effects at low exposure levels. The effects of compounds identified at higher concentrations in the tarragon extracts on other aquatic organisms were predicted using the admetSAR3.0 tool, and potential toxicity against numerous aquatic organisms was emphasized, particularly for cis-beta-ocimene, trans-beta-ocimene, and caryophyllene oxide. These findings emphasize the need for careful consideration of both the application dose and disposal practices of tarragon-based products.

## 1. Introduction

Scientific literature reveals that plant essential oils (EOs), due to their known diverse bioactive properties, play a significant role in phytochemistry, pharmacology, traditional medicine, cosmetics, food industry, and agricultural management. There also is a growing trend among consumers to favor natural products over conventional drugs [1] and a growing concern about the environmental impact and human health risks associated with synthetic pesticides that has driven a global movement toward the use of sustainable agricultural practices. Due to these factors, essential oils are produced on an industrial scale, which increases the likelihood of their release into the environment. It also underlines that the plant EOs market is experiencing strong global growth, and it has led to intensified research on naturally derived compounds. Despite their widespread use, the effects of these substances on ecosystems’ health are still poorly understood. Recent research has begun to investigate the potential toxicity of essential oils and plant-derived compounds on non-target species [2].

Tarragon (*Artemisia dracunculus*) is a perennial plant widely used in traditional medicine and the food industry. It has been recognized in aromatherapy, phytopharmacy, and traditional medicinal uses for its various beneficial effects, including antibacterial, antifungal, antiseptic, and antioxidant properties [3,4]. Furthermore, tarragon essential oil has proved its potential in pest management. Research studies indicated that tarragon EOs exhibited insecticidal activity [5,6,7], had antifungal properties when used in preventing post-harvest decay in orange fruits [8], and negatively affected the germination of weeds [9]. All these explain the growing tarragon EOs market size projected to reach over USD 1.1 billion by 2032 [10].

Plant essential oils are commonly viewed as natural and safe; however, when used improperly or in high concentrations, they can pose risks to both human health and the environment [11]. The growing market for tarragon EOs raises concerns about its potential environmental impacts, particularly on aquatic ecosystems. If tarragon EOs enter water bodies, they could potentially cause pollution and harm aquatic organisms. Direct research specifically addressing the entry of tarragon EOs into water bodies or their environmental fate in waterbodies could not be identified. Most existing studies focus on the chemical composition, bioactivity, or applied uses of tarragon EOs. Studies on essential oils and/or extracts of other species within the *Artemisia* genus reported toxicity for some aqueous organisms, with the toxic effects being usually observable at high concentrations [2,12].

Consequently, the aim of this study is to evaluate the effects of a tarragon hydrodistillate and a tarragon EO found on the Romanian market on aquatic ecosystems using a combination of experimental and computational approaches. The experimental approach considers the use of a growth inhibition test using *Lemna minor* to evaluate the potential ecotoxicity of tarragon EOs and a biochemical test to investigate the potential effects of the lowest volume of essential oil. The computational approach considers the admetSAR3.0 tool to predict the effects of the compounds found in higher concentrations in the investigated tarragon extracts on aquatic organisms. Our hypothesis is that the ecotoxicity of tarragon-based products on aquatic ecosystems is directly related to the concentration and chemical properties of volatile constituents, with essential oil causing more significant toxic effects than hydrodistillate.

## 2. Materials and Methods

### 2.1. Test Substances

Phytotoxicity assessments were conducted using two preparations of *Artemisia dracunculus* (tarragon): an aqueous extract and a commercially available essential oil (Aroma-Zone, Cabrières d’Avignon, France). To obtain the aqueous extract of tarragon, 100 g of freshly cut plant material was gently ground to avoid cellular degradation and then subjected to hydrodistillation at 105 °C using a Clevenger-type apparatus (Adrian Sistem Glassware, Bucharest, Romania). The process yielded two distinct phases: (i) a distillate composed of water and volatile essential oils and (ii) an aqueous solution formed by the condensation of steam within the distillation flask in contact with the plant material. These two solutions were separated using a separating funnel. The distillate phase was allowed to cool to room temperature and subsequently stored at +4 °C in airtight containers until further analysis [4]. The aqueous extract was evaluated across a dilution range from undiluted (1:1) to 1:1000, using Organization for Economic Cooperation and Development (OECD) standard growth medium as the diluent [13]. The essential oil was tested at volumes ranging from 0.1667 µL to 1000 µL per 10 mL of medium.

**Figure 1:**
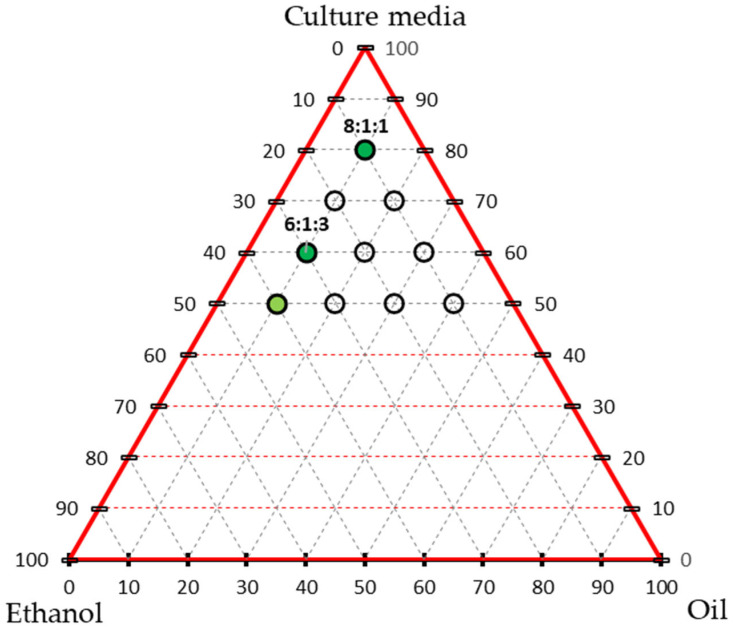
Triaxial graph representing the tested ratios of tarragon essential oil, ethanol, and culture medium. Each point (empty circles) corresponds to a tested formulation. Variants that formed stable emulsions (no phase separation) are marked in light and dark green. Dark-green points indicate the selected formulations for further testing on Lemna minor (with a medium:oil:ethanol ratio).

To improve the dispersion of the hydrophobic essential oil in the aqueous medium, supplementary tests were conducted to identify more homogeneous oil mixtures. Various ratios of essential oil, ethanol (as an emulsifying agent), and OECD medium were prepared. Mixtures containing 0% essential oil or ethanol, or less than 50% medium, were excluded. The remaining formulations were visually assessed for homogeneity, and the most stable emulsions—defined by the absence of visible oil droplets or phase separation—were selected for subsequent toxicity testing (Figure 1).

### 2.2. Test Organism and Culture Conditions

The test organism was *Lemna minor* L., a species widely recommended for aquatic phytotoxicity testing due to its simple structure, rapid growth, and sensitivity to contaminants. This free-floating aquatic plant forms small colonies of interconnected fronds, each measuring 2–5 mm in length, and has a high surface-to-volume ratio that facilitates direct contact with dissolved substances and floating oil droplets. Its ease of cultivation and sensitivity make it a practical bioindicator for assessing phytotoxic effects in aquatic environments [14,15].

The assay was conducted in accordance with the OECD Guideline 221: *Lemna* sp. Growth Inhibition Test. Each replicate consisted of ten fronds (two colonies with three fronds each and one colony with four fronds), cultivated in OECD standard growth medium. Test vessels were maintained under controlled conditions according to the OECD 221 standard [13].

### 2.3. Experimental Design

The experimental design followed the principles outlined in OECD Guideline 221 for the *Lemna* sp. growth inhibition test. All treatments were conducted in triplicate. Each replicate consisted of ten *L. minor* fronds, distributed as two colonies with three fronds each and one colony with four fronds. The fronds were exposed to test solutions for a duration of 7 days. To ensure effective contact between the essential oil droplets and the floating duckweed plants, small glass vials (2.5 cm in diameter and 4 cm in height) containing 10 mL of the test solution were used. This setup facilitated consistent exposure and minimized the loss or uneven distribution of the hydrophobic compounds within the treatment medium.

The following treatment groups were included in the study:

- Negative control (C−): OECD standard growth medium;
- Positive control (C+): 0.5% zinc chloride (ZnCl_2_) solution;
- Aqueous extract treatments (AE): serial dilutions of tarragon aqueous extract ranging from undiluted to 1:1000 dilution;
- Essential oil treatments (EO): a volume series of commercial tarragon essential oil, ranging from 0.1667 µL to 1000 µL per 10 mL medium, corresponding to oil-to-media volume ratios of approximately 0.0017% to 10% (*v*/*v*) and application rates of 0.034 to 204 µL/cm^2^, based on surface area;
- Homogenized oil mixture treatments (HOM): selected formulations of essential oil, ethanol, and medium that were identified as forming homogeneous mixtures during preliminary testing.

At the end of the exposure period, the following endpoints were recorded: number of colonies, number of green and chlorosed fronds, and the frond-to-colony ratio. These parameters were used to evaluate growth inhibition, construct dose–response curves, and determine the median effective dose (ED_50_) for the essential oil treatments. Where applicable, the data are presented in the figures as mean values, with error bars representing standard deviations calculated separately for each treatment group.

### 2.4. Biochemical Analyses

To assess potential sub-lethal effects at concentrations that did not result in visible phytotoxicity, the lowest tested volume of tarragon essential oil—0.1667 µL—was selected for biochemical evaluation. In parallel, an additional treatment was included consisting of 0.1667 µL of essential oil mixed with an equal volume (1:1, *v*/*v*) of ethanol to determine whether the presence of ethanol as a solubilizing agent influenced plant biochemical responses. The following physiological and biochemical parameters were measured:

- Fresh weight (FW);
- Photosynthetic pigments: chlorophyll a, chlorophyll b, total chlorophyll (a + b), and carotenoids (x + c);
- Primary metabolites: reducing sugars and total soluble proteins;
- Oxidative stress marker: catalase (CAT) enzymatic activity.

The fresh weight of *Lemna minor* fronds was determined by carefully removing the plants from the culture medium using forceps. Excess surface moisture was gently blotted with paper towels to avoid water bias. The fronds were then immediately weighed using an analytical balance with a precision of ±0.001 g.

Chlorophyll pigments were extracted from *Lemna minor* fronds using 80% acetone. Fronds were grinded using a tissue homogenizer and disperser (MiniBatch D-1, MICCRA GmbH, Heitersheim, Germany), and the homogenate was centrifuged at 18,000 g for 10 min. The resulting supernatant was collected for pigment analysis.

Aliquots of 100 µL of the supernatant were transferred to a 96-well microplate in triplicate, and absorbance was measured at 663 nm, 647 nm, and 470 nm using a microplate reader (Synergy H1, Agilent Technologies (formerly BioTek Instruments), Santa Clara, CA, United States). The concentrations of chlorophyll a (Chl a), chlorophyll b (Chl b), total chlorophyll (Chl a + b), and total carotenoids (xanthophylls + carotenes, Chl x + c) were calculated using the equations for 80% acetone extracts described by Lichtenthaler and Wellburn (1983) [16].

Primary metabolites—reducing sugars and total soluble proteins—were extracted from *Lemna minor* fronds by grinding in phosphate buffer (pH 7.2) using a tissue homogenizer and disperser (MiniBatch D-1, MICCRA). The homogenate was centrifuged under the same conditions as used for chlorophyll extraction, and the resulting supernatant was used for analysis.

Reducing sugars were quantified using the 3,5-dinitrosalicylic acid (DNS) method. In a 96-well microplate, 100 µL of each sample (in triplicate) was mixed with 50 µL of freshly prepared DNS reagent. The plate was incubated at 100 °C for 15 min to develop the colorimetric reaction, then cooled to room temperature. The absorbance of 3-amino-5-nitrosalicylic acid (ANS), the colored reaction product formed from the reduction of DNS reagent by reducing sugars, was measured at 575 nm using a microplate reader (Synergy H1, BioTek). Reducing sugar concentrations were calculated using a standard calibration curve generated with known concentrations of glucose.

Total soluble protein content was determined spectrophotometrically by measuring absorbance at 260 nm and 280 nm in triplicate using a microplate reader (Synergy H1, BioTek). Protein concentration was calculated using the Kalckar formula: Protein (mg/mL) = 1.55 × A280 − 0.76 × A260, which corrects for nucleic acid interference [17].

Catalase activity was measured spectrophotometrically using the phosphate buffer extract in microplates, with all samples analyzed in triplicate. The reaction mixture consisted of 40 µL of 0.2 M hydrogen peroxide (H_2_O_2_) and 60 µL of enzyme extract. The mixture was incubated for two separate time intervals, 1 min and 3 min, to assess substrate decomposition over the 2 min interval. After incubation, 200 µL of 5% potassium dichromate in glacial acetic acid was added to stop the enzymatic reaction and to colorimetrically quantify the residual hydrogen peroxide [18]. Samples were heated at 100 °C for 10 min to develop a chromic acetate complex, which was measured by absorbance at 570 nm using a microplate reader. Catalase activity was calculated by comparing the decrease in hydrogen peroxide concentration against a standard calibration curve prepared with known H_2_O_2_ concentrations.

### 2.5. Integrating Literature-Based Chemical Profiling with Dose–Response Analysis of Tarragon Essential Oil Components

In this study, two extracts of tarragon were considered, a commercial tarragon essential oil and a hydrodistillate obtained from dried tarragon, both found on the Romanian market. To explore the potential role of the individual compounds found in higher amounts in the investigated tarragon extracts on the aqueous toxicity of *Artemisia dracunculus*, we used published data on their chemical composition from a literature source [4]. The chemical compounds present in the two extracts were identified by using a gas chromatography–mass spectrometry method (GC-MS) using a Shimadzu QP 2010 Plus instrument (Columbia, SC, USA). The compounds that were identified in extracts in higher concentrations were: estragole (79.425% in tarragon EO and 82.093% in hydrodistilate), cis-β-ocimene (8.422% in tarragon EO and 1.567% in hydrodistillate), trans-β-ocimene (6.690% in tarragon EO and 2.089% in hydrodistillate), caryophyllene oxide (2.106% in tarragon hydrodistillate), limonene (3.135% in tarragon EO and 2.137% in hydrodistilate), eugenol (1.327% in tarragon hydrodistillate), eugenol acetate (1.869% in tarragon hydrodistilatte), methyl eugenol ether (0.378% in tarragon EO and 4.017% in hydrodistilate), and α-pinene (1.002% in tarragon EO and 0.371% in hydrodistillate) [4]. Based on these proportions, we estimated the quantity of each compound present in the volumes of essential oil used in our *Lemna minor* bioassays. For each tested volume, the estimated dose of individual compounds was calculated in milligrams. These values were then used to generate dose–response curves and calculate EC_50_ values, allowing for comparisons of the compounds’ potential contributions to the observed inhibitory effects. This approach enhances understanding of how the phytotoxicity of the essential oil may be linked to specific chemical constituents.

### 2.6. Prediction of the Effects of Compounds Identified in Tarragon Extracts on Aqueous Organisms

Aquatic toxicity refers to the potential of a chemical compound to cause harmful effects in aquatic environments, typically assessed through its impact on organisms such as fish, daphnia, or algae. admetSAR, which has been updated to version 3.0, is a freely available online tool designed to predict the absorption, distribution, metabolism, excretion, and toxicity (ADMET) profiles of chemical compounds [19,20,21]. This tool uses machine learning algorithms to generate robust and reliable predictions and includes a module related to environmental risk assessment. This module contains predictions of probabilities for effects related to aquatic toxicity, predictions that are based on models trained with data from the ECOTOX database and other relevant sources. In the present study, the admetSAR3.0 tool was used to obtain prediction probabilities of toxicity against fishes (*Bluegill sunfish*, *Rainbow trout, Sheepshead minnow*, and *Fathead minnow*), aquatic plants (P. subcapitata), crustaceans (*Daphnia magna*), and aquatic microorganisms (*Tetrahymena pyriformis*). These organisms were selected because they represent some of the most commonly used endpoints in experimental aquatic toxicology, enabling the development of reliable in silico models within the admetSAR 3.0 platform. The admetSAR3.0 tool has been widely used to predict the pharmacological profiles and ecotoxicity potentials of various chemicals [22,23].

### 2.7. Statistical Analysis

All data were statistically analyzed using PAST version 5.2.2 (Øyvind Hammer—University of Oslo, Oslo, Norway) [24]. The Shapiro–Wilk W test was employed to assess the normality of data distribution. Where parametric assumptions were met, one-way analysis of variance (ANOVA) was performed, with Levene’s test applied to confirm the homogeneity of variances. Post hoc comparisons were conducted using Tukey’s test. In cases where data did not meet the assumptions for parametric analysis, the Kruskal–Wallis test was used, followed by Dunn’s multiple comparison test for post hoc analysis. Dose–response curves and median effective dose (ED_50_) or concentration (EC_50_) values were generated using the AAT Bioquest^®^ online analysis tool [25]. Statistical significance was established at a threshold of *p* < 0.05.

## 3. Results

### 3.1. Effects of Aqueous Extract on Lemna Minor Growth

To assess the influence of the aqueous tarragon extract on *Lemna minor* growth, both frond number and colony structure were evaluated following exposure to various dilution levels. The number of fronds was expressed as a percentage relative to the negative control (C−) for comparability.

**Figure 2:**
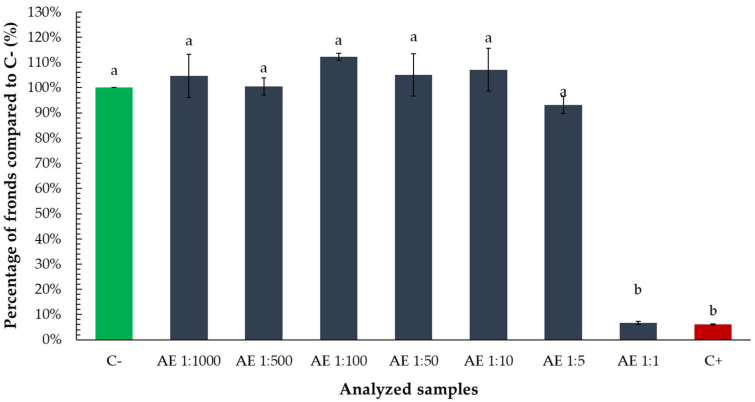
Percentage of *Lemna minor* fronds relative to the negative control (C−) following exposure to aqueous tarragon extract at various dilutions. Bars labeled with different letters represent treatments that are significantly different from each other (*p* < 0.05; one-way ANOVA followed by post hoc test). Treatments sharing the same letter are not significantly different.

A statistically significant reduction in frond number was observed only in the treatment with the undiluted extract, where frond percentage dropped to levels comparable to the positive control (zinc chloride 0.5%) (Figure 2).

**Figure 3:**
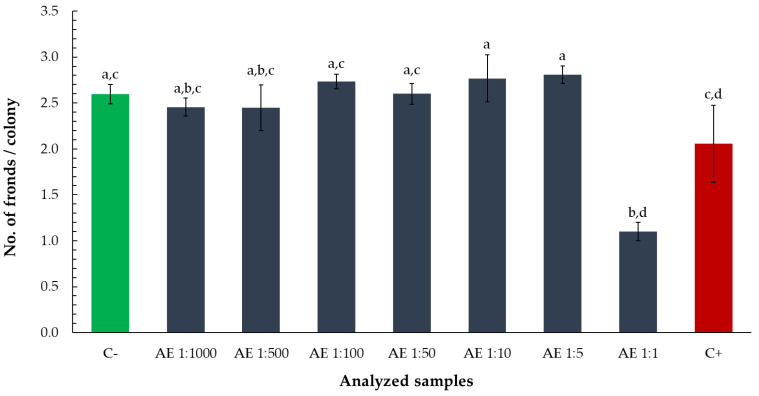
Number of fronds per colony of *Lemna minor* after exposure to aqueous tarragon extract at various dilutions. Bars labeled with different letters represent treatments that are significantly different from each other (*p* < 0.05; one-way ANOVA followed by post hoc test). Treatments sharing the same letter are not significantly different. If a bar has multiple letters (e.g., “ab”), it is not significantly different from any treatment sharing at least one of those letters.

At the same dilution level, the number of fronds per colony was also significantly lower than in the negative control and several higher dilutions, suggesting impaired vegetative propagation or colony development (Figure 3).

**Figure 4:**
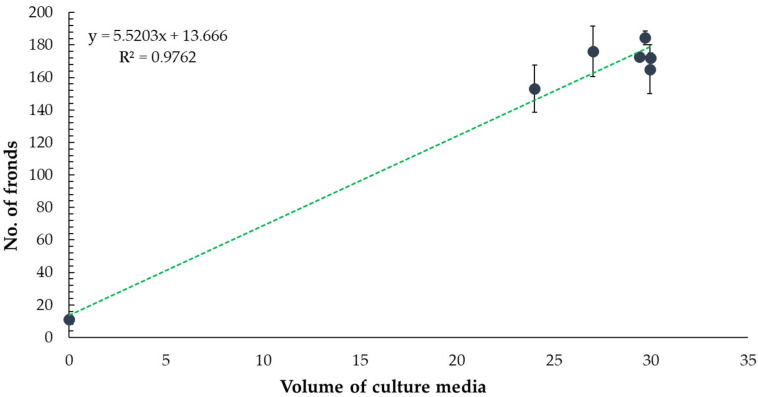
Effect of culture medium volume on the number of *Lemna minor* fronds. The graph shows a positive correlation between medium volume and frond number (R^2^ = 0.9762).

To explore whether the observed reduction in frond number was related to phytotoxicity or nutrient limitation, we examined the effect of culture medium volume on frond production. The data showed a positive correlation (R^2^ = 0.9762) between medium volume and frond number, suggesting that the lower growth seen with the undiluted extract may be influenced by reduced medium availability rather than direct toxicity (Figure 4).

These findings suggest that the aqueous extract of tarragon does not exhibit inherent phytotoxicity toward *Lemna minor* under the tested conditions.

### 3.2. Effects of Tarragon Essential Oil on Lemna Minor Growth

To assess the influence of tarragon essential oil on *Lemna minor* growth, the number of fronds and colony structure were evaluated following exposure to a range of oil volumes. Frond number was expressed as a percentage relative to the negative control (C−) to allow for direct comparison across treatments.

**Figure 5:**
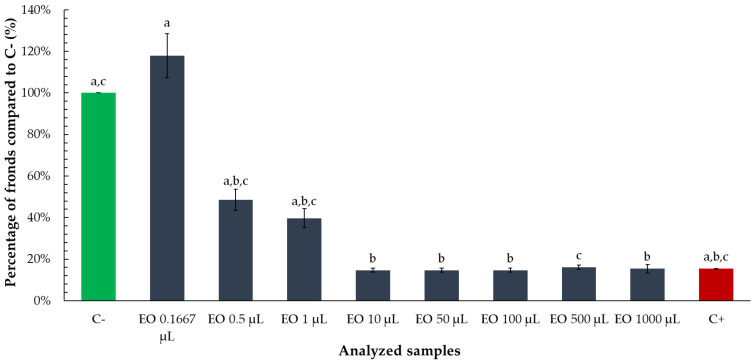
Percentage of *Lemna minor* fronds relative to the negative control (C−) following exposure to tarragon essential oil at various volumes. Bars labeled with different letters represent treatments that are significantly different from each other (*p* < 0.05; one-way ANOVA followed by post hoc test). Treatments sharing the same letter are not significantly different. If a bar has multiple letters (e.g., “ab”), it is not significantly different from any treatment sharing at least one of those letters.

A statistically significant reduction in frond percentage was observed at volumes ranging from 10 to 1000 µL, indicating a clear inhibitory effect of the essential oil at these concentrations. Lower volumes of 1 µL and 0.5 µL also resulted in reduced frond numbers, with a 40–50% decrease compared to the negative control, although the effect was less pronounced than at higher volumes. Interestingly, the lowest tested volume, 0.1667 µL, did not cause any reduction in frond number. In fact, plants exposed to this volume exhibited approximately 18% more fronds than the negative control (Figure 5), possibly reflecting a stimulatory effect consistent with hormesis. In this dose-dependent response, secondary metabolites in essential oils act as hormetins—chemical agents that at low or intermittent levels activate defense and resilience pathways, whereas higher or prolonged exposure typically leads to cellular damage [26].

**Figure 6:**
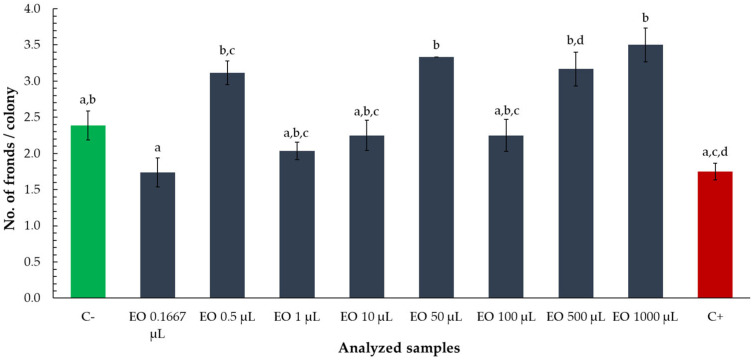
Number of fronds per colony of *Lemna minor* after exposure to tarragon essential oil at various volumes. Bars labeled with different letters represent treatments that are significantly different from each other (*p* < 0.05; one-way ANOVA followed by post hoc test). Treatments sharing the same letter are not significantly different. If a bar has multiple letters (e.g., “ab”), it is not significantly different from any treatment sharing at least one of those letters.

To evaluate potential alterations in colony structure, the number of fronds per colony was assessed following exposure to various volumes of tarragon essential oil. Although no statistically significant differences were observed between the treatments and the negative control (C−), some trends emerged across the tested volumes (Figure 6).

The fronds per colony ratio was lower than the negative control for volumes of 0.1667, 1, 10, and 100 µL, though these values remained higher than those observed in the positive control (zinc chloride). In contrast, volumes of 0.5, 50, 500, and 1000 µL yielded higher frond-to-colony ratios than the negative control, suggesting that, while not statistically distinct, certain oil concentrations may subtly influence colony morphology. However, the overall lack of significant deviation from the negative control indicates that tarragon essential oil did not markedly affect colony structure under the tested conditions.

**Figure 7:**
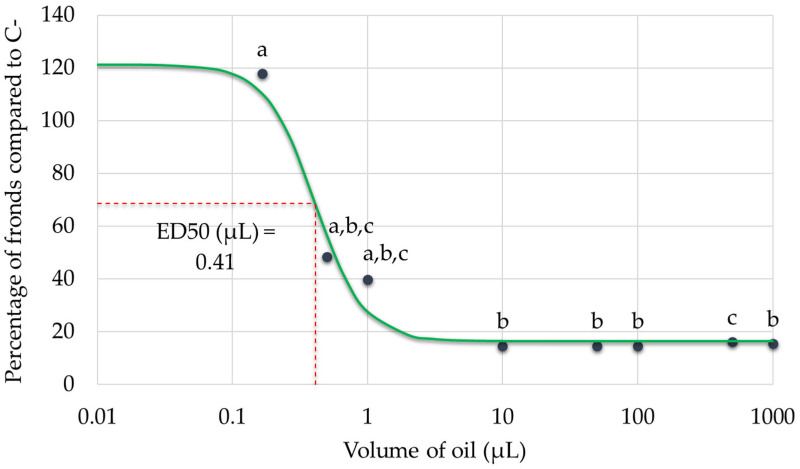
Dose–response curve showing the effect of increasing volumes of tarragon essential oil on Lemna minor growth. The effective dose required to reduce frond number by 50% (ED_50_) is indicated on the curve. Black dots represent experimental data points. The green continuous line indicates the fitted dose–response model, while the red dashed lines serve as visual guides to identify the ED_50_ value, marked along the curve. Data points labeled with different letters represent treatments that are significantly different from each other (*p* < 0.05; one-way ANOVA followed by post hoc test). Treatments sharing the same letter are not significantly different. If a bar has multiple letters (e.g., “a,b,c”), it is not significantly different from any treatment sharing at least one of those letters.

The dose–response relationship between tarragon essential oil volume and *Lemna minor* growth inhibition was successfully established by plotting the percentage of fronds relative to the negative control against increasing oil volumes. The resulting curve demonstrated a clear, dose-dependent inhibitory effect. From the fitted model, the effective dose required to reduce frond number by 50% (ED_50_) was estimated at approximately 0.41 µL, which equates to 0.0041% (*v*/*v*) and around 0.084 µL/cm^2^. This quantitative benchmark highlights the phytotoxic potential of tarragon essential oil on aquatic plants and supports its relevance in assessing environmental impact (Figure 7).

The results demonstrate that tarragon essential oil exerts a dose-dependent inhibitory effect on *Lemna minor* growth, with significant reductions in frond number observed at volumes as low as 0.5 µL. The dose–response relationship enabled the calculation of an ED_50_ value, indicating the volume at which frond growth is reduced by 50%. Notably, the lowest tested volume (0.1667 µL) did not inhibit growth and instead slightly stimulated frond production, suggesting a possible hormetic effect at minimal concentrations. While some variations in frond-to-colony ratios were observed across treatments, these differences were not statistically significant relative to the negative control, indicating that essential oil primarily affects total frond production rather than colony structure. Overall, these findings highlight the phytotoxic potential of tarragon essential oil at higher volumes and support its possible application in plant-based bioassays or ecological risk assessment.

### 3.3. Biochemical Response to a Non-Growth-Inhibitory Volume of Essential Oil

To elucidate the metabolic responses of *Lemna minor* to a sub-inhibitory volume of tarragon essential oil, a comprehensive analysis of key biochemical parameters was undertaken. Specifically, the concentrations of photosynthetic pigments (chlorophylls a and b, total chlorophyll, and carotenoids), primary metabolites (reducing sugars and total soluble proteins), and the activity of the antioxidant enzyme catalase were quantified. These measurements were performed following exposure to the lowest essential oil volume that did not produce growth inhibition. This investigation aims to provide insight into the subtle biochemical alterations induced by non-toxic treatment levels, thereby enhancing the understanding of the plant’s adaptive and stress-related responses beyond growth metrics.

**Figure 8:**
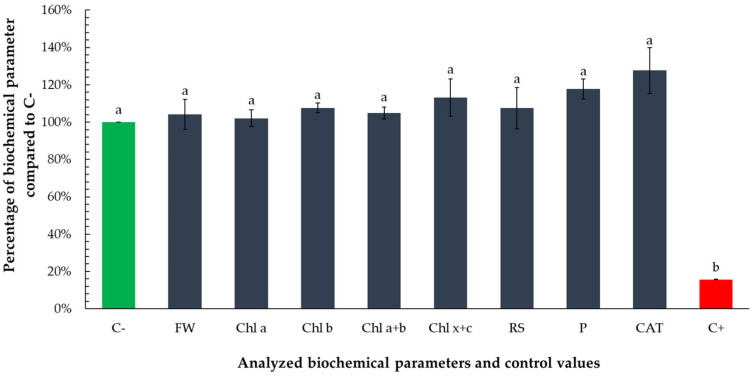
Biochemical response of *Lemna minor* to the non-growth-inhibitory volume (0.1667 µL) of tarragon essential oil. Bars represent the mean values of the analyzed biochemical parameters—photosynthetic pigments (chlorophyll a, chlorophyll b, total chlorophyll, and carotenoids), reducing sugars, total soluble proteins, and catalase activity—expressed as percentages relative to the negative control (C−). Values for the negative and positive control are included as means of percentages of all analyzed parameters. Bars labeled with different letters represent treatments that are significantly different from each other (*p* < 0.05; one-way ANOVA followed by post hoc test). Treatments sharing the same letter are not significantly different.

Exposure to the non-growth-inhibitory volume (0.1667 µL) of tarragon essential oil did not induce any statistically significant changes in the measured biochemical parameters, compared to the negative control. However, all analyzed parameters were statistically different from positive control (Figure 8).

All analyzed parameters exhibited slightly elevated mean values relative to the negative control, suggesting subtle physiological modulation rather than stress-induced alteration. Notably, carotenoid concentration (Chl x + c) increased by approximately 13%, total soluble protein content by 18%, and catalase activity by 28%. These moderate increases, though not statistically significant, may indicate a mild stimulatory or priming effect at sub-toxic concentrations.

The increase in carotenoids could reflect enhanced photoprotective capacity or early antioxidant responses, while elevated protein content may signal a boost in general metabolic activity. The increase in catalase activity, an enzyme central to the detoxification of hydrogen peroxide, may suggest the initiation of low-level oxidative signaling, potentially associated with hormesis—a biphasic response to low-dose exposure. Overall, these observations support the conclusion that the tested volume does not impair physiological function and may even promote slight metabolic activation in *Lemna minor*.

### 3.4. Biological and Biochemical Effects of Selected Essential Oil–Ethanol Mixtures

To evaluate the biological relevance and potential toxicity of more homogeneously dispersed formulations, selected mixtures of tarragon essential oil and ethanol were tested on *Lemna minor*. Due to the hydrophobic nature of essential oils, application in aqueous environments often results in poor solubility and surface-layer accumulation, leading to uneven exposure. The inclusion of ethanol, a water-miscible solvent, was intended to improve the distribution of the essential oil throughout the culture medium, allowing for more uniform contact with plant tissues.

This experimental design also aimed to determine whether any increased biological or biochemical effects observed in the presence of oil–ethanol mixtures were attributable to the essential oil itself or to ethanol-related toxicity. To this end, ethanol was included as a separate control treatment. Both frond growth and key biochemical parameters—photosynthetic pigment content, concentrations of primary metabolites, and catalase activity—were measured to assess the impact of these formulations on the physiology of *Lemna minor*.

**Figure 9:**
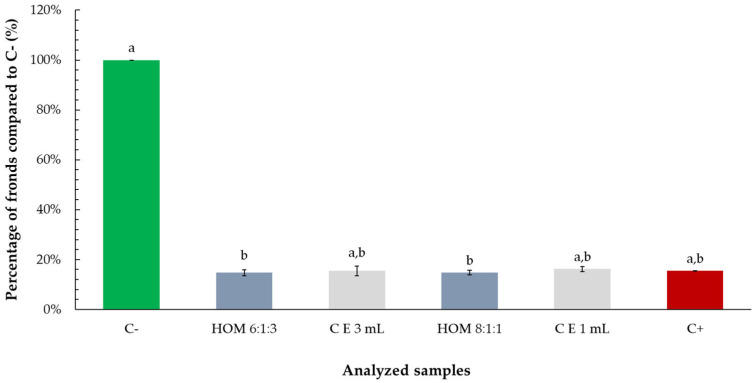
Percentage of *Lemna minor* fronds relative to the negative control (C−) following exposure to tarragon essential oil mixtures with ethanol at various ratios (culture media:oil:ethanol). Bars labeled with different letters represent treatments that are significantly different from each other (*p* < 0.05; one-way ANOVA followed by post hoc test). Treatments sharing the same letter are not significantly different. If a bar has multiple letters (e.g., “a,b”), it is not significantly different from any treatment sharing at least one of those letters.

Both essential oil–ethanol mixtures (ratios 6:1:3 and 8:1:1) and ethanol alone reduced the percentage of *Lemna minor* fronds by approximately 20%, compared to the negative control. However, statistical analysis revealed that only the reductions caused by the mixtures were significant, while the effect of ethanol alone was not statistically different from the control. These findings suggest that although ethanol may contribute somewhat to growth inhibition, the presence of essential oil in the mixtures is primarily responsible for the observed statistically significant decrease in frond proliferation (Figure 9).

**Figure 10:**
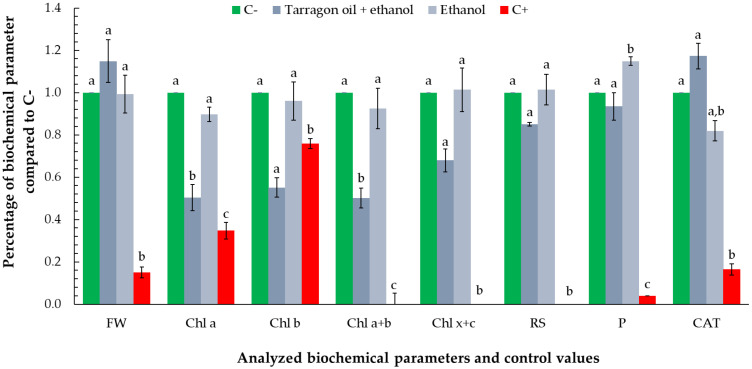
Biochemical response of *Lemna minor* to a mixture of 0.1667 µL of essential oil combined with an equal volume of ethanol (1:1, *v*/*v*), and an ethanol control of the same volume. Bars labeled with different letters represent treatments that are significantly different from each other (*p* < 0.05; one-way ANOVA followed by post hoc test). Treatments sharing the same letter are not significantly different. If a bar has multiple letters (e.g., “ab”), it is not significantly different from any treatment sharing at least one of those letters.

Exposure to the essential oil–ethanol mixture (1:1) and ethanol control resulted in no statistically significant differences compared to the negative control across most measured biochemical parameters. However, notable exceptions were observed for chlorophyll a and total chlorophyll (a + b), where the oil–ethanol mixture induced approximately a 50% reduction relative to the control. Conversely, protein concentration in the ethanol treatment was about 20% higher than the negative control, while the oil–ethanol mixture showed protein levels comparable to the control. Catalase activity displayed contrasting responses: it was elevated by approximately 20% in the oil–ethanol mixture treatment, potentially indicating an induction of oxidative stress, whereas ethanol alone caused nearly a 20% decrease in catalase activity relative to the negative control (Figure 10).

These differential responses suggest that while ethanol alone may modulate protein synthesis without triggering oxidative stress, the essential oil component in the mixture could be responsible for oxidative challenges reflected in increased catalase activity and reduced chlorophyll content.

### 3.5. Integrating Literature-Based Chemical Profiling with Dose–Response Analysis of Tarragon Essential Oil Components

To better understand the observed phytotoxicity, we used published data on the chemical composition of tarragon essential oil to estimate the doses of major constituents present in the tested volumes, enabling dose–response analysis and EC_50_ determination for each compound [4]. According to the referenced composition study of *Artemisia dracunculus* extracts, the most abundant constituents in tarragon essential oil were estragole, cis-β-ocimene, trans-β-ocimene, and limonene. These compounds were reported as the dominant volatile components based on their percentage abundance, suggesting they may significantly contribute to the biological activity of the essential oil (Table 1).

**Figure 11:**
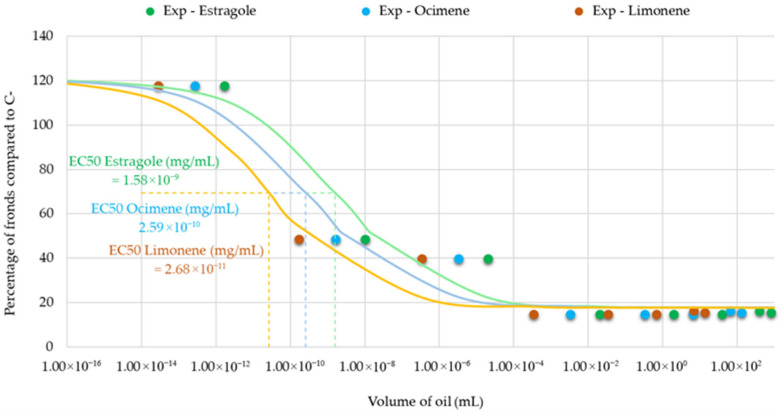
Dose–response curves for the major constituents of Artemisia dracunculus essential oil—estragole (p-allylanisole), (Z/E)-β-ocimene, and limonene—based on estimated concentrations corresponding to tested oil volumes. EC_50_ values were calculated for each compound to evaluate their individual contributions to the observed phytotoxic effects.

The results showed that the EC_50_ values for the individual compounds varied according to their initial concentrations, reflecting their differing contributions to the inhibitory effects (Figure 11).

This correlation suggests that the toxicity of the essential oil may not be solely attributed to the mere presence of active constituents but also to their estimated exposure levels, which are influenced by physicochemical properties such as water solubility. However, further experimental validation using individual compounds would be necessary to confirm this relationship. Notably, estragole and limonene, which possess moderate water solubility, may partially dissolve into the aqueous phase, enabling broader plant surface contact and greater bioavailability. In contrast, (Z)- and (E)-β-ocimene, which are insoluble in water, are likely to exert effects primarily through direct contact with undissolved oil droplets, limiting their systemic distribution.

### 3.6. Prediction of the Effects of Compounds Identified in Tarragon Extracts on Aqueous Organisms

Predictions obtained using the admetSAR3.0 tool for toxicity of compounds identified in higher concentrations in tarragon extracts against aquatic organisms are revealed in Table 2.

Data presented in Table 2 expose that all the identified compounds may be toxic for some investigated aquatic organisms, with caryophyllene oxide being the compound revealing the highest aquatic toxicity. A published study indicates that caryophyllene oxide, a major constituent of *Artemisia campestris* essential oil, has shown toxic effects on aquatic organisms such as brine shrimp [30]. Among the aquatic organisms considered, *Tetrahymena pyriformis* seems to be most affected by all the compounds found in tarragon extracts.

## 4. Discussion

Environmental exposure levels of tarragon EOs can vary widely depending on its source of use (agriculture, cosmetics, industry), but no direct data on these exposure levels could be identified. Despite the lack of quantitative data, environmental exposure to tarragon extracts is presumed to occur due to their widespread use.

*Lemna minor* is widely used as a model organism in ecotoxicological and physiological studies. Numerous peer-reviewed articles report the effects of various chemicals, including pesticides, heavy metals, pharmaceuticals, plant extracts, and essential oils, on its growth, pigment content, enzymatic activity, and general physiology. To the best of our knowledge, this is the first study assessing the effects of tarragon hydrodistillate and EO on *Lemna minor*. The results obtained reveal that the aqueous extract of *Artemisia dracunculus* does not exhibit inherent phytotoxicity toward *Lemna minor*, most probably due to the fact that it contains low levels of potentially toxic compounds [4,31]. The absence of phytotoxic effects indicates that tarragon-derived aqueous solutions are unlikely to pose ecological risks to non-target aquatic organisms in environments where such exposure might occur. Published research presents mixed findings regarding the effects of aqueous plant extracts on *Lemna* species. While some studies report inhibitory effects on growth, pigment content, or enzymatic activity, suggesting phytotoxicity [32], others demonstrate neutral or even stimulatory effects [33], indicating that effects may vary depending on the plant source, extract concentration, and exposure duration.

Regarding the tarragon essential oil, the results obtained indicate that it exerts a dose-dependent inhibitory effect on the growth of *Lemna minor,* primarily influencing total frond proliferation rather than colony architecture. It is not an unexpected result, as it was already shown that tarragon EOs can be used as biopesticides due to their phytotoxic properties based on their content on various compounds, which can inhibit plant growth and development [4,34]. Similar results have been recorded for the essential oil extracted from several other plants [35,36]. Therefore, this effect can be seen as the synergistic and individual actions of the diverse chemical compounds within EOs that lead to the dose-dependent inhibition of *Lemna minor*’s growth and its characteristic reduction in frond proliferation. Literature data also reveal that usually, plant EOs exhibit higher toxicity than plant aqueous extracts [2].

*Lemna minor* is widely recognized as a model organism for assessing metabolic responses to various environmental stressors due to its sensitivity and rapid growth [37]. In the present study, the measured biochemical parameters, including photosynthetic pigments, reducing sugars, soluble proteins, and catalase activity, exhibited slightly elevated mean values relative to the negative control. These modest increases suggest subtle physiological modulation rather than pronounced stress-induced alteration. The absence of significant deviations from baseline values indicates that the tested volume of tarragon EO does not impair the physiological functions of *Lemna minor* and may, in fact, promote slight metabolic activation. These findings support the conclusion that low-level exposure to the essential oil may lead to mild stimulation of biochemical activity without triggering toxicity.

Certain compounds identified in tarragon EO, most notably estragole and methyl eugenol, have demonstrated toxicity potential, particularly at higher concentrations. This effect, even at low aqueous concentrations, is due to their lipophilicity and ability to interact with cell membranes. Another compound found in tarragon extracts is ocimene, a volatile, hydrophobic monoterpene hydrocarbon with low water solubility and low environmental persistence due to its high vapor pressure and tendency to degrade in the atmosphere [38]. It suggests that ocimene evaporates or partitions into the air or organic phases rather than staying dissolved in water for long periods. However, aquatic toxicity from ocimene can still occur because it may temporarily accumulate at the water surface as an oily film or droplets, and aquatic organisms inhabiting surface microlayers can be directly exposed via contact or ingestion [39].

While the solubility of the tarragon EO is low, the effect of what little dissolves, or is finely dispersed, can be toxic. Consequently, the present study also considered assessment of the potential toxicity of the compounds identified in tarragon hydrodistillate and EO against other aquatic organisms. Predicted data reveal that all the compounds may exert at least moderate toxicity against aquatic microorganisms, crustaceans, and fishes, with caryophyllene oxide being the compound revealing the highest aquatic toxicity. To the best of our knowledge, no studies have directly reported aquatic toxicity at environmentally relevant concentrations of tarragon EO. Taking into account that tarragon EO contains bioactive constituents like estragole, methyl eugenol, and α-pinene and the structural similarities of these compounds to known terpenoids, revealing human [40] and environmental [41] toxicity, the potential toxicity against aquatic organisms cannot be neglected.

The results obtained in this study are in agreement with published data revealing that some essential oils and extracts have shown no observable toxicity toward aquatic organisms, or such effects occurred only at elevated concentrations; however, others have been found to be toxic, even at concentrations lower than those permitted by international regulatory standards [2]. Further studies assessing long-term exposure and higher concentration ranges may be warranted to fully characterize the environmental safety profile of these tarragon extracts.

## 5. Conclusions

Aqueous tarragon extract showed no phytotoxic effects on *Lemna minor*, with significant growth reduction observed only in the undiluted extract, likely due to nutrient limitation rather than toxicity.

Essential oil demonstrated dose-dependent phytotoxicity, inhibiting growth at volumes of 0.5 µL and higher, with an ED_50_ value confirming its inhibitory potential. Mixtures of essential oil and ethanol, formulated to enhance homogeneity and exposure, caused growth inhibition comparable to the same volume (1 mL) of essential oil alone, indicating that improved dispersion did not increase toxicity beyond that of the pure oil at equivalent concentrations.

Biochemical analyses indicated that while the essential oil alone induced minimal changes, the more homogeneous oil–ethanol mixtures led to significant alterations in chlorophyll content, protein concentration, and oxidative stress markers, suggesting greater physiological impact due to improved dispersion and uptake.

Taken together, these findings demonstrate that tarragon essential oil is phytotoxic to *Lemna minor*, especially at higher volumes, whereas the aqueous extract remains non-toxic under the tested conditions.

The phytotoxicity of tarragon essential oil is influenced by the exposure levels of its major constituents. Estragole, the compound identified in the highest amount in tarragon extracts, exhibits limited water solubility but can form azeotropic mixtures facilitating its partitioning into the aqueous phase, thereby enhancing its bioavailability and uptake by plants [42]. In contrast, ocimene, another compound found in tarragon extracts, is nearly insoluble in water and remains largely in the oil phase. As a result, it exerts phytotoxic effects primarily through direct contact with undissolved oil droplets rather than via aqueous-phase uptake, being considered toxic to aquatic life with long lasting effects [43].

This study presents several limitations. First, it includes only *Lemna minor* as a representative aquatic species in the experimental approach, while other aquatic species are considered solely through computational predictions. This narrow taxonomic scope may not adequately reflect the broader ecological impact of tarragon essential oil. Second, the experiments were conducted under controlled laboratory conditions, which may not fully capture the complexity of natural environments, including variable physicochemical factors and biotic interactions. Third, the study focuses on acute toxicity, potentially overlooking the effects of chronic or low-dose exposure that are more representative of environmental realities. Future research should address these limitations by incorporating a wider range of test organisms, including species from aquatic, terrestrial, and microbial ecosystems. In addition, long-term exposure studies are necessary to better assess ecological risks associated with repeated or sustained exposure to the essential oil. A broader biochemical analysis across multiple concentrations should also be included to provide a more comprehensive understanding of sublethal and concentration-dependent effects and to help elucidate potential mechanisms of toxicity. Finally, investigations into the environmental fate of tarragon essential oil—particularly its degradation pathways and the toxicity of its metabolites in aquatic and/or soil environments—are essential for a comprehensive understanding of its ecotoxicological profile.

## Figures and Tables

**Table 1:** Major constituents of tarragon essential oil, their relative concentrations (as reported in Pujicic et al., 2025 [4]), and water solubility values retrieved from PubChem for estragole [27], ocimene [28], and limonene [29].

Compound	Concentration in Essential Oil	Solubility in Water
Estragole	79.425%	178 mg/L
Ocimene (total)	15.110%	Insoluble
Limonene	3.153%	7.57 mg/L

**Table 2:** Predictions of the probabilities that chemical compounds found in higher concentrations in tarragon extracts exhibit aquatic toxicity: green cells reveal low toxicity potential, yellow cells reveal mean toxicity potential, and red cells reveal high toxicity potential.

Compound/Aquatic Organism Toxicity	*P. subcapitata* Toxicity	*D. magna* Toxicity	*Fathead minnow* Toxicity	*Bluegill sunfish* Toxicity	*Rainbow trout* Toxicity	*Sheepshead minnow* Toxicity	*T. pyriformis* Toxicity
estragole	0.638	0.735	0.622	0.593	0.826	0.690	0.985
α-Pinene	0.752	0.887	0.800	0.563	0.788	0.725	0.982
cis-beta-ocimene	0.813	0.891	0.874	0.602	0.738	0.705	0.990
trans-beta-ocimene	0.812	0.893	0.874	0.605	0.738	0.702	0.990
limonene	0.766	0.804	0.790	0.414	0.579	0.59	0.970
Eugenol methyl ether	0.482	0.513	0.362	0.515	0.702	0.616	0.968
Eugenol acetate	0.631	0.621	0.575	0.610	0.702	0.639	0.955
Eugenol	0.552	0.491	0.328	0.606	0.606	0.472	0.940
Caryophyllene oxide	0.883	0.950	0.929	0.651	0.822	0.770	0.991

## Data Availability

All the data are available in the manuscript.
